# Yeast Culture Enhances Production Performance in Late-Lactation Dairy Cows by Reshaping Rumen Microbiota and Metabolic Pathways

**DOI:** 10.3390/vetsci13040336

**Published:** 2026-03-31

**Authors:** Jiahui Yu, Zhi Dou, Can Wang, Shendong Zhou, Huimin Shi, Hongzhu Zhang, Hongze Wang, Nana Ma, Xiangzhen Shen, Guangjun Chang

**Affiliations:** 1 Ministry of Education Joint International Research Laboratory of Animal Health and Food Safety, College of Veterinary Medicine, Nanjing Agricultural University, Nanjing 210095, China; 2 Angel Yeast Co., Ltd., Yichang 443000, China

**Keywords:** dairy cow nutrition, lactation performance, metabolomics, oxidative stress, rumen microbiome, volatile fatty acids, yeast culture

## Abstract

Yeast culture (YC), a complex functional feed additive containing fermentation metabolites, has demonstrated potential in dairy production systems. However, its mechanistic effects on rumen function and host metabolism in lactating dairy cattle warrant further investigation. This study evaluated the impacts of YC supplementation on production performance, systemic antioxidant status, and rumen function in late-lactation Holstein cows. Fourteen multiparous Holstein cows (body weight 655 ± 28 kg; days in milk 270.4 ± 1.6 d) were randomly allocated into two groups, a control group (CON, basal diet) and a YC-supplemented group (YC, basal diet + 50 g/d YC), in a 28-day feeding trial. YC supplementation significantly increased dry matter intake (*p* < 0.01), nutrient digestibility (*p* < 0.01 for DM, CP, EE, NDF, and ADF), and milk yield (*p* < 0.05) compared to CON. Systemic antioxidant capacity was enhanced, as evidenced by elevated serum superoxide dismutase activity (*p* < 0.01) and total antioxidant capacity (*p* < 0.05). Rumen fermentation was improved with higher concentrations of total volatile fatty acids (*p* < 0.01) and microbial protein (*p* < 0.01) and reduced ammonia nitrogen levels (*p* < 0.01). Macrogenomic analysis revealed a YC-mediated restructuring of the rumen microbiota, characterized by an increased relative abundance of Firmicutes and decreased Bacteroidota. Untargeted metabolomic profiling identified significant alterations in rumen metabolite profiles, with differential metabolites enriched in pyrimidine metabolism and vitamin digestion and absorption pathways. These results indicate that YC supplementation improves production performance in late-lactation dairy cows through multi-faceted mechanisms involving rumen microbial community modulation and metabolic pathway activation, ultimately enhancing nutrient utilization and metabolic efficiency.

## Simple Summary

Yeast culture shows promise as a feed additive for supporting dairy cow productivity. In this study, we examined how dietary supplementation with yeast culture affects late-lactation Holstein cows. Our results demonstrated that yeast culture supplementation significantly improved feed intake (+3.1%), nutrient digestibility (dry matter +6%, crude protein +20%), and milk yield (+5.75 kg/d) compared with the control group (*p* < 0.05). These production benefits were associated with improved rumen function, characterized by a higher production of volatile fatty acids and a more stable microbial community. Collectively, our findings substantiate the use of yeast culture as a practical nutritional strategy to enhance production efficiency in dairy farming during the critical late-lactation period. Data were analyzed using *t*-tests, with statistical significance set at *p* < 0.05.

## 1. Introduction

High-yielding dairy cows require substantial energy to support milk synthesis, a demand typically met by increasing the proportion of concentrates in their diet [1,2]. However, this practice can disrupt rumen health and predispose cows to subacute ruminal acidosis (SARA) [2]. The diet used in this study represents a moderate-concentrate ration (16.82% crude protein [CP], 52.4% crude fiber [CF]; Table 1). The experimental cows were in late lactation (mean days in milk [DIM] ≈ 270), a stage associated with a lower risk of SARA due to reduced fermentable carbohydrate intake and enhanced fiber digestion capacity [3]. Therefore, this study focuses on the effects of yeast culture on rumen function and production performance under typical feeding conditions.

Various feed additives, including direct-fed microbials and prebiotics, have been extensively investigated as strategies to optimize rumen function [3,4]. Among these, yeast culture (YC)—a fermentation product of Saccharomyces cerevisiae—has garnered significant interest due to its complex profile of bioactive metabolites, such as peptides, organic acids, and enzymes, which collectively modulate rumen microbiota and fermentation processes [5]. In ruminants, meta-analytical evidence consistently demonstrates that YC supplementation (1–10 g/d) enhances dry matter intake, milk yield, and nutrient digestibility while concurrently stabilizing ruminal pH and volatile fatty acid (VFA) profiles [6,7]. Furthermore, recent findings indicate that YC may improve systemic antioxidant capacity in dairy goats [8]. Nevertheless, the majority of existing research has centered on early- or mid-lactation cows, leaving the impact of YC on late-lactation animals—a physiological phase characterized by altered nutrient requirements and an increased susceptibility to negative energy balance [9]—relatively under-investigated. Despite these well-documented production responses, the underlying mechanisms remain incompletely understood, and effects on specific ruminal parameters are sometimes inconsistent across studies [10,11]. This variability highlights a persistent knowledge gap regarding YC’s precise mode of action. Moreover, most research has focused on early- or mid-lactation cows. However, the effects on late-lactation animals—a phase characterized by distinct metabolic and physiological priorities, including a reduced nutrient demand for milk production, an increased risk of negative energy balance, and altered immune function [9]—have been relatively underexplored.

The efficacy of yeast-based additives is fundamentally linked to their interaction with the rumen ecosystem. As the primary site of microbial fermentation, the rumen harbors a complex and dynamic consortium of bacteria, archaea, protozoa, and fungi. The composition and functional output of this microbiota are critical for feed digestion, volatile fatty acid (VFA) production, and overall host homeostasis [12]. A prevailing hypothesis is that YC modulates this microbial community, thereby stabilizing ruminal pH, improving nutrient digestion and nitrogen utilization efficiency, and mitigating acidosis risk [13,14]. Proposed mechanisms include the stimulation of fibrolytic microbial activity, leading to enhanced fermentation with increased VFA production and reduced ammonia nitrogen [15], as well as direct or indirect effects on lactate concentration and pH dynamics [16]. However, a comprehensive and causal understanding of how YC specifically reshapes the ruminal microbiome at both taxonomic and functional levels, and how these microbial changes translate into measurable host benefits, is still lacking.

The advent of high-throughput omics technologies offers an unprecedented opportunity to address this knowledge gap. Metagenomics enables a comprehensive, gene-centric profiling of the entire microbial community, moving beyond taxonomic identification to infer its functional potential. Concurrently, untargeted metabolomics provides a global profile of the small-molecule metabolites within a biological sample, offering a direct snapshot of the rumen’s biochemical state. While each approach is powerful independently, the integrated application of metagenomics and metabolomics to investigate feed additive interventions in dairy cows remains relatively limited. This multi-omics strategy can effectively bridge microbial genetic potential with actual metabolic output, thereby enabling the construction of robust mechanistic hypotheses.

We hypothesized that dietary YC supplementation would enhance the production performance of late-lactation dairy cows by: (1) enriching populations of fiber-degrading bacteria (e.g., Firmicutes); (2) promoting ruminal propionate production; and (3) upregulating pyrimidine metabolism pathways, thereby facilitating enhanced nutrient utilization and systemic antioxidant capacity.

Accordingly, the objective of this study was to evaluate the effects of YC supplementation on production performance, nutrient digestibility, serum antioxidant status, and ruminal fermentation, while further elucidating the underlying mechanisms through an integrated multi-omics approach—specifically leveraging metagenomic and metabolomic analyses.

## 2. Materials and Methods

### 2.1. Animals, Ethics, and Experimental Design

This study was conducted from June to July 2023 at the Modern Dairy Bengbu Ranch (Bengbu, Anhui Province, China). All animal handling and experimental procedures were reviewed and approved by the Animal Care and Use Committee of Nanjing Agricultural University (Approval No. NJAU.PZ.20231120174) and were conducted in strict compliance with the Laboratory Animal Regulations of the Ministry of Science and Technology, China.

Fourteen healthy Holstein cows in late lactation were selected based on the following criteria: parity 2–3, body weight 655 ± 28 kg, days in milk (DIM) 270.4 ± 1.6 d, milk yield 34.5 ± 5.9 kg/d, and dry matter intake (DMI) 23.51 ± 0.53 kg/d. Animals were blocked by parity and days in milk and then randomly allocated to either the control or experimental group, with seven cows per treatment: (1) the control group (CON), which received a basal total mixed ration (TMR); and (2) the yeast culture group (YC), which received the basal TMR supplemented with 50 g/head/day of a commercial yeast culture product (Angie’s Yeast Co., Ltd., Yichang, Hubei, China). This dosage was selected based on the manufacturer’s recommendation and previous studies reporting beneficial effects with similar supplementation levels [17]. The detailed formulation and nutritional composition of the basal diet are provided in Table 1.

Following a 7-day acclimation period to the new environment and diets, the formal experimental feeding phase lasted for 28 consecutive days. Cows were fed the TMR three times daily at 06:00, 13:00, and 18:00. The YC supplement was top-dressed and thoroughly mixed into the ration during the morning (06:00) and evening (18:00) feedings. All animals had ad libitum access to fresh water and were managed under identical housing and environmental conditions. Daily feed intake and individual milk yield were recorded throughout the entire experimental period.

### 2.2. Sample Collection and Analysis of Production Performance

#### 2.2.1. Feed and Fecal Sampling and Analysis

Samples of the total mixed ration (TMR) were collected on days 23, 24, and 25 of the experimental period. The samples were dried in a forced-air oven at 65 °C for 48 h, ground to pass through a 1 mm sieve, and stored in airtight containers for subsequent chemical analysis. Fecal samples were collected via rectal palpation at 08:00 and 16:00 on the day after the experimental period (day 29). One aliquot of fresh feces was immediately preserved in brown bottles containing 10% (*v*/*v*) hydrochloric acid for subsequent nitrogen determination. A separate aliquot was dried at 65 °C to a constant weight for the analysis of other conventional nutrients.

#### 2.2.2. Determination of Dry Matter Intake and Nutrient Digestibility

Dry matter intake (DMI) was calculated daily by recording the total weight of feed offered and the weight of orts collected the following morning prior to feeding. The apparent total-tract digestibility of nutrients was determined using acid-insoluble ash (AIA) as an internal inert marker. The AIA content in feed and fecal samples was determined according to the International Organization for Standardization (ISO) method 5985:2002 [18,19]. The apparent digestibility coefficient for each nutrient was calculated using the following standard formula: Apparent digestibility (%) = [1 − (AIAfeed × Nutrientfeces)/(AIAfeces × Nutrientfeed)] × 100.

#### 2.2.3. Chemical Analysis

All feed and fecal samples were analyzed in duplicate. The contents of dry matter (DM, method 934.01), crude protein (CP, method 990.03; N × 6.25), and ether extract (EE, method 920.39) were determined according to the official methods of the Association of Official Analytical Chemists (AOAC, 2023) [20]. The contents of neutral detergent fiber (NDF) and acid detergent fiber (ADF) were analyzed according to the procedures described by Van Soest et al. (1991) [18], using heat-stable amylase for NDF analysis and expressed on an ash-free basis.

#### 2.2.4. Milk Yield and Composition Analysis

Individual milk yield was recorded daily. All cows were milked three times per day at 07:00, 13:30, and 20:00. On day 29, the yield from each milking was measured using a graduated flow meter (Model JHF-G17, Yulin, Sichuan, China). Milk samples were collected from each milking session, pooled proportionally by volume, preserved with potassium dichromate, and stored at 4 °C. The pooled samples were submitted to the Dairy Herd Improvement (DHI) laboratory of Nanjing Weigang Dairy Co. for the analysis of milk composition, including fat, protein, and lactose percentages, milk urea nitrogen (MUN) content, and somatic cell count (SCC).

### 2.3. Blood Sample Collection and Biochemical Analysis

On day 29 of the experimental period, blood samples were collected aseptically from the coccygeal vein of each cow prior to the morning feeding. The samples were allowed to clot at room temperature for 30 min and then centrifuged at 3500× *g* for 15 min at 4 °C to separate the serum. The serum was aliquoted into sterile microtubes and stored at –20 °C until subsequent biochemical analysis.

Commercial colorimetric assay kits (Nanjing Jiancheng Bioengineering Institute, Nanjing, China) were used to quantify serum oxidative stress biomarkers following the manufacturer’s protocols. The methods for SOD, MDA, CAT, T-AOC, and GSH were based on established protocols [21,22,23,24,25]. The biomarkers analyzed included malondialdehyde (MDA, kit no. A003-1), superoxide dismutase (SOD, kit no. A001-3), catalase (CAT, kit no. A007-1-1), total antioxidant capacity (T-AOC, kit no. A015-2-1), and reduced glutathione (GSH, kit no. A006-2-1). Additionally, serum biochemical parameters, including glucose (GLU), albumin (ALB), alanine aminotransferase (ALT), aspartate aminotransferase (AST), and blood urea nitrogen (BUN), were determined using a fully automated clinical chemistry analyzer (Beckman Coulter, Brea, CA, USA).

### 2.4. Rumen Fluid Sampling, Fermentation Parameters, and Omics Sample Preparation

Rumen fluid was collected from each cow before the morning feeding on the day after the experimental period (day 29) using an oro-ruminal stomach tube connected to a vacuum pump. The pH of the freshly collected fluid was measured immediately using a calibrated portable pH meter (Hanna Instruments, Woonsocket, RI, USA).

For the analysis of fermentation parameters, a representative portion of the rumen contents was filtered through four layers of sterile cheesecloth. The filtrate was processed as follows: one aliquot was stored at −20 °C for the subsequent determination of ammonia nitrogen (NH_3_-N) and microbial crude protein (MCP) concentrations. Another aliquot was prepared for volatile fatty acid (VFA) analysis by mixing 5 mL of rumen fluid with 1 mL of 25% (*w*/*v*) metaphosphoric acid. The mixture was held at –20 °C overnight, then centrifuged at 12,000× *g* for 10 min at 4 °C. The resulting supernatant was filtered through a 0.22 μm polyethersulfone membrane syringe filter prior to gas chromatographic analysis.

The phenol–hypochlorite colorimetric method was used to determine NH_3_-N levels [26]. MCP concentration was quantified according to the Coomassie Brilliant Blue G-250 dye-binding method. Individual VFAs (acetate, propionate, butyrate, isobutyrate, valerate, and isovalerate) along with total VFAs (TVFA) were quantified via gas chromatography using a GC-14B system (Shimadzu Corporation, Kyoto, Japan) fitted with a flame ionization detector (FID) and a capillary column (30 mm × 0.32 mm i.d., 0.25 μm film thickness) following previously described procedures [27]. The column oven temperature program was 110 °C held for 2 min, then ramped to 180 °C at 10 °C/min, and maintained at 180 °C for 5 min. The injector and detector temperatures were both maintained at 180 °C. High-purity nitrogen was used as the carrier gas at a constant flow rate of 30 mL/min.

For subsequent metagenomic and metabolomic analyses, approximately 5 mL of unfiltered rumen contents were immediately transferred into two pre-labeled, sterile cryovials, snap-frozen in liquid nitrogen, and stored at –80 °C until further processing.

### 2.5. DNA Extraction, Metagenomic Sequencing, and Bioinformatics

#### 2.5.1. DNA Extraction and Quality Control

Microbial genomic DNA was extracted from the frozen rumen content samples using the E.Z.N.A.^®^ Stool DNA Kit (Omega Bio-tek, Norcross, GA, USA) in accordance with the manufacturer’s protocol. Extracted DNA was quantified and assessed for purity using a NanoDrop 2000 spectrophotometer (Thermo Fisher Scientific, Waltham, MA, USA). DNA integrity was checked through electrophoresis on 1.0% (*w*/*v*) agarose gels.

#### 2.5.2. Library Preparation and Sequencing

Sequencing libraries for metagenomic shotgun sequencing were prepared and sequenced by Shanghai Biozeron Biological Technology Co., Ltd. (Shanghai, China). Briefly, for each sample, 1 μg of qualified genomic DNA was randomly fragmented to an average size of approximately 450 bp using a Covaris S220 Focused-ultrasonicator (Covaris, Woburn, MA, USA). Sequencing libraries were prepared following the standard Illumina TruSeq Nano DNA library preparation protocol and sequenced on an Illumina NovaSeq 6000 platform (Illumina, San Diego, CA, USA) in a paired-end 150 bp (PE150) configuration.

#### 2.5.3. Sequence Data Preprocessing

Quality control and adapter removal of raw sequencing reads were performed using Trimmomatic v0.36 [17], applying the following settings: SLIDINGWINDOW:4:15 MINLEN:50. To remove potential host contamination, the resulting high-quality reads were aligned to the bovine reference genome (assembly ARS-UCD1.2) using the BWA-MEM algorithm (v0.7.17) [28]. Reads that mapped to the host genome were discarded. The remaining host-free reads were retained for all subsequent bioinformatic analyses.

#### 2.5.4. Taxonomic Profiling and Diversity Analysis

For taxonomic classification, the clean reads from each sample were analyzed using Kraken2 (v2.1.2) [29] against a custom-built database. This database was constructed from all complete bacterial, archaeal, viral, fungal, and protozoan genome sequences downloaded from the NCBI RefSeq database (release date: 9 December 2022). The relative abundance of taxa at each phylogenetic rank (from domain to species) was subsequently estimated and refined using Bracken (v2.6.2) [30]. Microbial α-diversity was assessed using the Shannon and Simpson indices. β-diversity was evaluated by calculating Bray–Curtis dissimilarities based on species-level abundance profiles, followed by visualization via principal coordinate analysis (PCoA).

#### 2.5.5. Metagenomic Assembly, Gene Catalog Construction, and Quantification

De novo assembly of the host-free reads from each sample was performed independently using MEGAHIT (v1.1.1-2-g02102e1) [31] with the parameter-min-contig-len 500. Open reading frames (ORFs) were predicted from the assembled contigs using MetaProdigal (v2.6.3) [32]. A non-redundant gene catalog was constructed by clustering all predicted protein-coding sequences from all samples at 95% nucleotide sequence identity and 90% coverage using CD-HIT (v4.8.1) [33]. The abundance of each gene in each sample was quantified by mapping the clean reads back to this non-redundant gene catalog using BWA-MEM (v0.7.17). Gene abundance was normalized and expressed as transcripts per million (TPM).

#### 2.5.6. Functional Annotation

The non-redundant gene catalog was functionally annotated by searching against the Kyoto Encyclopedia of Genes and Genomes (KEGG) database (release 1 January 2023) using KofamScan (v1.2.0) [34] with default parameters to obtain KEGG Orthology (KO) and pathway annotations.

Carbohydrate-active enzyme (CAZy) annotations were obtained by comparing the gene catalog against the CAZy database (version 8, accessed on 1 October 2023) using the dbCAN2 meta server [35]. The abundance of a specific KEGG pathway or CAZy family in a given sample was calculated by summing the TPM values of all genes annotated to that functional category.

### 2.6. Ruminal Metabolomic Profiling by UPLC-MS/MS

#### 2.6.1. Metabolite Extraction

The frozen rumen fluid samples were thawed on ice. For metabolite extraction, a 100 μL aliquot of each sample was mixed with 400 μL of pre-chilled methanol (LC-MS grade) through vigorous vortexing for 30 s. The mixture was kept on ice for 5 min, followed by centrifugation at 15,000× *g* for 10 min at 4 °C. From the resulting supernatant, 300 μL was collected and combined with 270 μL of LC-MS grade water, yielding a final methanol concentration of approximately 53% (*v*/*v*). The diluted extract was centrifuged once more using identical conditions (15,000× *g*, 10 min, 4 °C), and the resultant supernatant was carefully pipetted into a glass insert placed inside a certified LC-MS injection vial for further analysis.

#### 2.6.2. UPLC-MS/MS Analysis

Untargeted metabolomic analysis was carried out on an ultra-high-performance liquid chromatography system (Vanquish, Thermo Fisher Scientific, Bremen, Germany) interfaced with a high-resolution quadrupole-Orbitrap mass spectrometer (Q Exactive™ HF, Thermo Fisher Scientific, Bremen, Germany) at Biozeron Co., Ltd. (Shanghai, China). Chromatographic separation was achieved on a Hypesil Gold C18 column (100 mm × 2.1 mm i.d., 1.9 μm particle size; Thermo Fisher Scientific) maintained at 40 °C. The mobile phase composition differed between ionization modes:

Positive ion mode: (A) 0.1% (*v*/*v*) formic acid in water; (B) methanol.

Negative ion mode: (A) 5 mM ammonium acetate in water, pH adjusted to 9.0 with ammonium hydroxide; (B) methanol. For both modes, the flow rate was set at 0.2 mL/min with the following linear gradient elution program: 2% B (0–1.5 min), increased linearly to 100% B (1.5–12.0 min), held at 100% B (12.0–14.0 min), returned to 2% B (14.0–14.1 min), and re-equilibrated at 2% B (14.1–17.0 min). The injection volume was 2 μL. The mass spectrometer was operated in polarity switching mode to acquire data in both positive and negative electrospray ionization (ESI) modes within a single injection. Key source parameters were as follows: spray voltage, ±3.2 kV; capillary temperature, 320 °C; sheath gas flow rate, 40 arbitrary units (arb); auxiliary gas flow rate, 10 arb. Full MS scans were acquired over a mass range of *m*/*z* 70–1050 with a resolution of 60,000 (at *m*/*z* 200). Data-dependent MS/MS scans (dd-MS^2^) were triggered for the top 10 most intense ions per scan cycle using a stepped normalized collision energy of 20, 40, and 60 eV.

#### 2.6.3. Data Processing and Metabolite Identification

The raw data files (.raw) were imported and processed using Compound Discoverer software (version 3.1, Thermo Fisher Scientific). The workflow included peak detection, alignment across all samples, retention time correction, and peak area integration. Key processing parameters were set as follows: retention time tolerance, 0.2 min; mass tolerance, 5 ppm; intensity tolerance, 30%; signal-to-noise (S/N) threshold, 3; and minimum peak intensity, 1 × 10^5^.

The peak intensity matrix was normalized by the total spectral intensity of each sample to correct for systematic variations. Putative metabolite identification was performed by matching the accurate mass (±5 ppm) and MS/MS fragmentation spectra against three databases: the online mzCloud database (Thermo Fisher Scientific), an in-house mzVault library, and a custom MassList containing known rumen metabolites.

#### 2.6.4. Statistical Analysis and Pathway Enrichment

The normalized and log-transformed peak intensity data were subjected to multivariate statistical analysis using the metaX R software package [36]. Principal component analysis (PCA) was performed for an unsupervised overview of sample clustering. Orthogonal partial least squares discriminant analysis (OPLS-DA) was employed to enhance separation between the predefined experimental groups and to pinpoint metabolites that contributed most to group discrimination.

The selection of differential metabolites was based on the following combined criteria: a variable importance in projection (VIP) score > 1.0 derived from the OPLS-DA model, a fold change (FC) ≥ 2.0 or ≤0.5, and a *p*-value < 0.05 from a two-tailed Student’s *t*-test (with or without false discovery rate adjustment, as specified in results). Pathway enrichment analysis of the identified differential metabolites was conducted by mapping their KEGG identifiers to the KEGG pathway database (https://www.genome.jp/kegg/ accessed on 1 September 2023) using hypergeometric tests.

For metabolite identification, confidence levels were assigned according to the Metabolomics Standards Initiative (MSI) guidelines: metabolites identified by accurate mass and MS/MS spectral matching with public databases (mzCloud, mzVault) were classified as level 2. MS/MS spectral matching was performed with a mass tolerance of 5 ppm. Batch effect correction was performed using quality control (QC) sample-based normalization. QC samples were injected every 10 samples throughout the analytical run to monitor system stability and correct for potential signal drift. Missing values were handled by filtering out features with >50% missingness and imputing remaining missing values with half the minimum value. Although internal standards were not employed, metabolite abundances were normalized by total spectral intensity to correct for sample-specific variations.

### 2.7. Integrated Multi-Omics Correlation Analysis

To elucidate potential mechanistic linkages among the rumen microbiome, its metabolic output, and host physiological responses, an integrative Spearman’s rank correlation analysis was performed. Key variables from the following four data layers were included:

Microbiome: The relative abundance of microbial genera that was identified as significantly differential between the CON and YC groups.

Microbial Function: The abundance (in TPM) of key differential carbohydrate-active enzyme (CAZy) families.

Metabolome: The relative levels of significant differential metabolites identified from the ruminal metabolomic profiling.

Host Phenotype: Crucial performance and physiological indices, including daily milk yield, dry matter intake, ruminal propionate concentration, and serum superoxide dismutase (SOD) activity.

Pairwise Spearman’s rank correlation coefficients (ρ) were calculated for all selected variables across all samples. To focus on robust biological associations, only strong correlations (|ρ| > 0.70) with statistical significance (adjusted *p*-value < 0.05, using the Benjamini–Hochberg false discovery rate method) were retained for downstream analysis and interpretation. The resulting correlation network was visualized using Cytoscape software (version 3.9.1) [37].

### 2.8. Statistical Analysis

Data are presented as the mean ± standard error of the mean (SEM). Statistical analyses for production performance, serum biochemical/oxidative stress parameters, and ruminal fermentation characteristics were performed using SPSS Statistics software (version 25.0; IBM Corp., Armonk, NY, USA).

Normality of data distribution was evaluated with the Shapiro–Wilk test, and homogeneity of variances was checked using Levene’s test. For variables meeting the assumptions of normality and homogeneity of variance, differences between the CON and YC groups were evaluated using an independent two-sample Student’s *t*-test. For variables violating these assumptions, the non-parametric Mann–Whitney U test was applied as an alternative.

Differences were considered statistically significant at *p* < 0.05. A trend toward significance was noted for 0.05 ≤ *p* < 0.10.

## 3. Results

### 3.1. Feed Intake and Nutrient Apparent Digestibility

The effects of yeast culture (YC) supplementation on feed intake and the apparent digestibility of nutrients are presented in Table 2. Compared to the CON group, the YC group exhibited a significantly higher feed intake (+3.1%, *p* = 0.02). Furthermore, the apparent digestibility of dry matter (DM), crude protein (CP), ether extract (EE), neutral detergent fiber (NDF), and acid detergent fiber (ADF) were all significantly increased in the YC group (*p* < 0.01).

### 3.2. Milk Yield and Composition

Milk production performance data are summarized in Table 3. Dietary supplementation with YC resulted in a significantly higher average daily milk yield compared to the CON group (*p* < 0.05). Regarding milk composition, the lactose percentage was significantly increased (*p* = 0.05) in the YC group, while no significant differences were observed in milk fat percentage, milk protein percentage, milk urea nitrogen content, or somatic cell count (*p* > 0.05).

### 3.3. Serum Biochemical and Antioxidant Parameters

As shown in Table 4, YC supplementation significantly altered several serum biomarkers. The antioxidant capacity was enhanced, as indicated by a highly significant increase in superoxide dismutase (SOD) activity (*p* < 0.01) and a significant increase in total antioxidant capacity (T-AOC) (*p* < 0.05). For metabolic and health indicators, the YC group showed highly significantly higher glucose (GLU) levels (*p* < 0.01) and blood urea nitrogen (BUN) concentrations (*p* < 0.01), alongside significantly elevated albumin (ALB) levels (*p* < 0.01). Alanine aminotransferase (ALT) activity was significantly lower in the YC group (*p* < 0.05). No significant changes were detected in malondialdehyde (MDA), catalase (CAT), or aspartate aminotransferase (AST) levels between the groups (*p* > 0.05).

### 3.4. Rumen Fermentation Characteristics

Rumen fermentation parameters are detailed in Table 5. Supplementation with YC led to a highly significant decrease in the ammonia–nitrogen (NH_3_-N) concentration (*p* < 0.01). Concurrently, the microbial crude protein (MCP) content was highly significantly increased (*p* < 0.01). The concentration of lactic acid was significantly lower (*p* < 0.01), whereas the concentrations of acetic acid, propionic acid, and butyric acid were all significantly higher in the YC group (*p* < 0.05 for acetic acid; *p* < 0.01 for propionic and butyric acid). Consequently, the acetic acid-to-propionic acid ratio was highly significantly lower (*p* < 0.01), and the total volatile fatty acid (TVFA) concentration was highly significantly greater (*p* < 0.01) compared to the CON group.

### 3.5. Rumen Metagenomic Sequencing and Microbial Diversity

**Figure 1:**
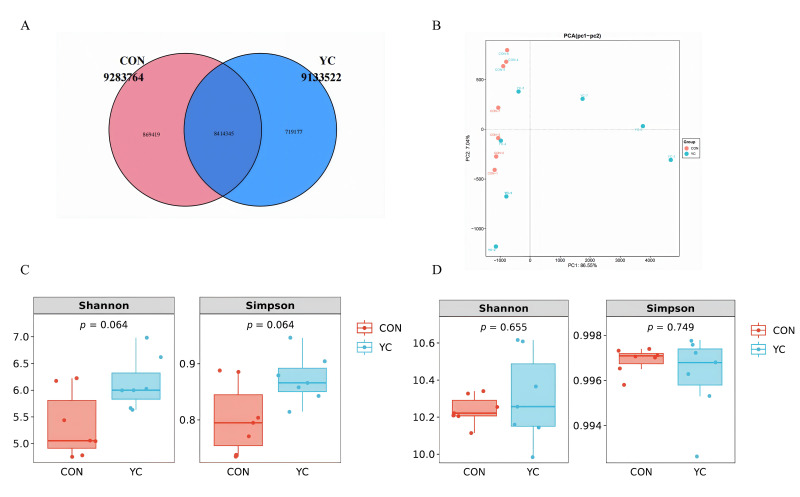
Effects of dietary yeast culture (YC) supplementation on rumen microbial diversity in late-lactation dairy cows. (**A**) Venn diagram illustrating the number of unique and shared microbial genes between the control (CON) and YC-supplemented groups. (**B**) Beta diversity analysis (Bray–Curtis dissimilarity) visualized via principal coordinate analysis (PCoA). (**C**) Alpha diversity indices at the gene level (observed features and Shannon index). (**D**) Alpha diversity indices at the species level (observed features and Shannon index). Data are presented as mean ± SEM *(n* = 7 per group). Statistical significance (*p*-value) is indicated on each panel.

Sequencing of rumen samples yielded a total of 1,641,880,048 raw reads. After quality control and host contamination removal, 1,595,522,184 high-quality reads (average 113,965,870 per sample) were obtained for subsequent analysis. The assembly of these reads produced 11,744,258 contigs (average N50 = 1254 bp). The achieved sequencing depth provided a high genome coverage for the majority of the rumen microbial community, enabling robust downstream analysis. A non-redundant gene catalog was constructed, revealing 8,414,345 genes common to all samples (Figure 1A), with 869,419 and 719,177 genes unique to the CON and YC groups, respectively.

Principal component analysis (PCA) based on gene abundance showed that the first two principal components (PC1 and PC2) explained 86.55% and 7.04% of the total variance, respectively (Figure 1B). Similar clustering patterns were observed in the PCoA plot based on Bray–Curtis distance (Figure 1C), with no clear separation between the two groups. The analysis of α-diversity indices revealed a trend toward increased microbial diversity at the genus level in the YC group, as indicated by higher Shannon and Simpson indices (*p* = 0.06, Figure 1D). However, no significant change was observed at the species level (*p* > 0.05, Figure 1D).

### 3.6. Rumen Microbiota Composition and Taxonomic Shifts

**Figure 2:**
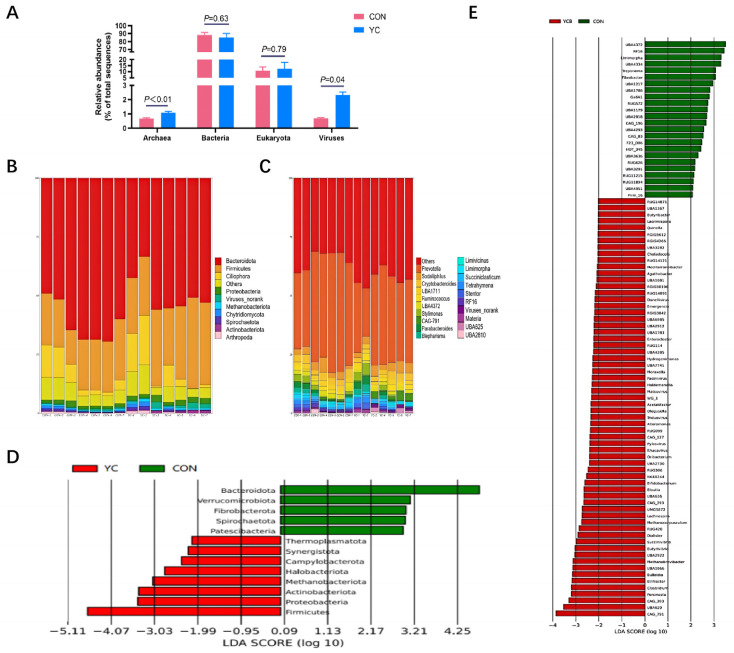
Effects of dietary yeast culture (YC) supplementation on rumen microbial composition in late-lactation dairy cows. (**A**) Domain level. (**B**) Phylum level. (**C**) Genus leve. (**D**) LEfSe analysis at the phylum level. (**E**) LEfSe analysis at the genus level.l. Taxa with an average relative abundance of less than 1% across all samples are grouped as “Others.” Data are presented as mean ± SEM (n = 7 per group). Statistical significance (*p*-value) is indicated on each panel.

Taxonomic annotation against the NR database showed that bacteria dominated the rumen microbiota (87.86% average relative abundance), followed by eukaryotes (10.46%), viruses (1.27%), and archaea (0.83%) (Figure 2A). YC supplementation highly significantly increased the relative abundance of archaea and viruses (*p* < 0.01). At the phylum level, Bacteroidota, Firmicutes, Ciliophora, Proteobacteria, Viruses_norank, and Methanobacteriota were the dominant taxa (Figure 2B,C). Differential abundance analysis (Table 6) indicated that YC supplementation highly significantly increased the relative abundance of Firmicutes and Viruses_norank (*p* < 0.01), and significantly increased Methanobacteriota (*p* < 0.05). In contrast, the relative abundances of Bacteroidota and Spirochaetota were highly significantly decreased (*p* < 0.01), while Patescibacteria and Verrucomicrobiota were significantly lower (*p* < 0.05). At the genus level, Prevotella and UBA4372 were highly significantly reduced in the YC group (*p* < 0.01). LEfSe analysis (LDA ≥ 1, *p* < 0.05) further identified distinct microbial biomarkers. At the phylum level, 13 taxa were differentially abundant (Figure 2D). At the genus level, 70 genera showed significant differences, with 12 genera (e.g., CAG_791, Clostridium, Butyricoccus) exhibiting an LDA score > 3 in the YC group compared to CON (Figure 2E).

### 3.7. Functional Profiling of the Rumen Microbiome

**Figure 3:**
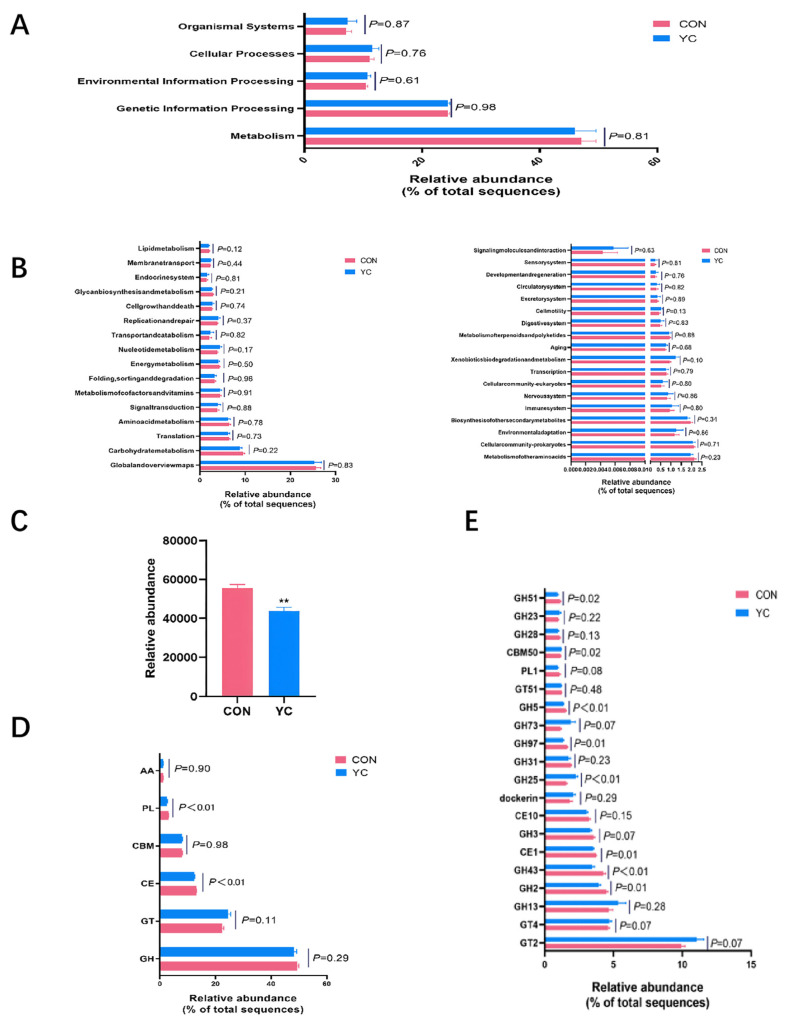
Effects of dietary yeast culture (YC) supplementation on predicted functional profiles of the rumen microbiome in late-lactation dairy cows. (**A**) Kyoto Encyclopedia of Genes and Genomes (KEGG) pathway classification at level 1. (**B**) KEGG pathway classification at level 2. (**C**–**E**) Carbohydrate-active enzyme (CAZyme) classification at the (**C**) overall, (**D**) class, and (**E**) family levels. Data are presented as mean ± SEM (n = 7 per group). Statistical significance (*p*-value) is indicated on each panel. Asterisks denote statistical significance (** *p* < 0.01).

Functional potential was assessed via KEGG and CAZy annotations. KEGG analysis revealed that “Metabolism” (48.41%) was the most abundant level 1 pathway, followed by “Genetic Information Processing” (23.82%), though no significant inter-group differences were observed at this level (*p* > 0.05, Figure 3A). Level 2 and level 3 pathways are detailed in Figure 3B.

**Figure 4:**
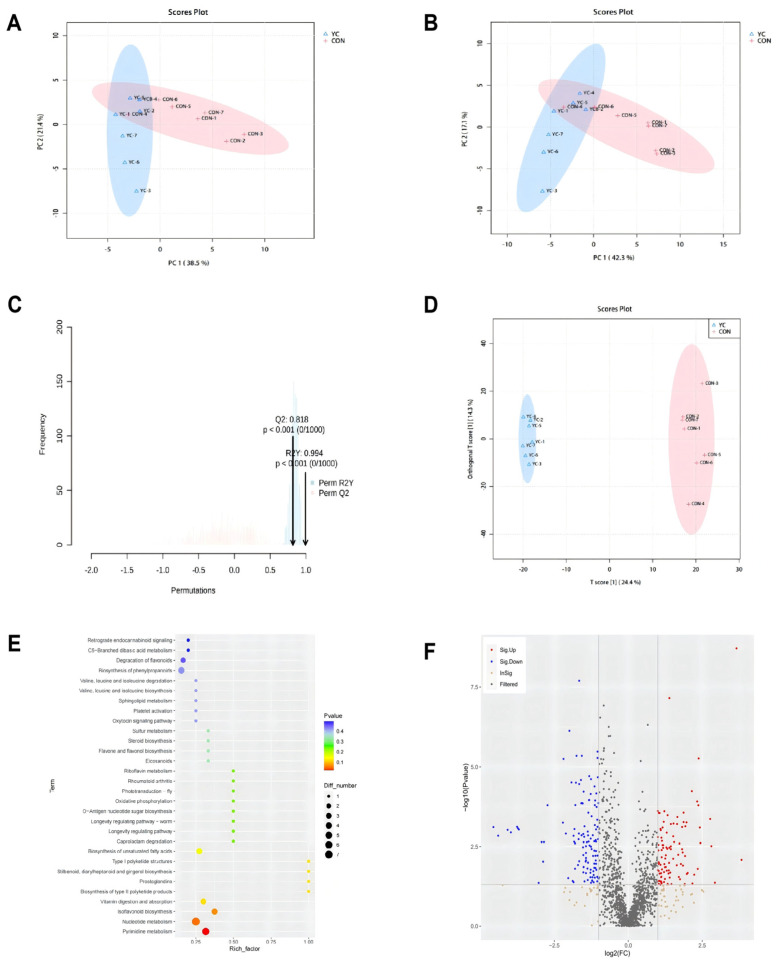
Effects of dietary yeast culture (YC) supplementation on the rumen fluid metabolome in late-lactation dairy cows. (**A**,**B**) Principal component analysis (PCA) score plots derived from untargeted metabolomics data in (**A**) negative and (**B**) positive ionization modes. (**C**) Validation plot of the orthogonal partial least squares discriminant analysis (OPLS-DA) model via permutation testing (200 iterations). (**D**) OPLS-DA score plot showing separation between the CON and YC groups. (**E**) Volcano plot displaying differentially abundant metabolites (criteria: fold change > 1.5 or <0.67, *p* < 0.05). Metabolites upregulated in the YC group are shown in red, downregulated in blue, and non-significant in gray. (**F**) Bubble chart of Kyoto Encyclopedia of Genes and Genomes (KEGG) pathways significantly enriched by the differential metabolites (*p* < 0.05). Bubble size represents the number of metabolites mapped to the pathway; color intensity represents the enrichment significance [−log_10_(*p*-value)].

Annotation against the CAZy database identified 346 carbohydrate-active enzyme (CAZyme) genes. Glycoside hydrolases (GHs, 49.19%) were most abundant, followed by Glycosyl Transferases (GTs, 23.06%) (Figure 4A). Overall, the total abundance of CAZymes was significantly lower in the YC group (*p* < 0.05). Specifically, at the class level, polysaccharide lyases (PLs) and carbohydrate esterases (CEs) were highly significantly reduced (*p* < 0.01, Figure 4D). At the family level, YC supplementation significantly increased the relative abundance of GT2, dockerin, GH73, and GH25 enzyme genes, while significantly decreasing GH2, GH43, CE1, GH97, GH5, GH3, and GH51 (*p* < 0.05, Figure 4E).

### 3.8. Rumen Metabolomic Profile

Rumen fluid metabolites were profiled using ultra-performance liquid chromatography–mass spectrometry (UPLC-MS) in widely targeted metabolomics mode. Samples were analyzed separately in positive and negative ionization modes. Following signal filtering, missing value imputation, and data normalization, a total of 1610 metabolite peaks were detected (984 in negative mode, 626 in positive mode). Of these, 479 metabolites were successfully annotated (182 in negative mode, 297 in positive mode).

The annotated metabolites were classified into major chemical categories including 376 lipids and lipid-like molecules, 201 organic acids and derivatives, 143 organic heterocyclic compounds, 96 benzenoids, 61 organic oxides, 60 nucleosides/nucleotides/analogs, 59 phenylpropanoids and polyketides, 22 organic nitrogen compounds, and 9 alkaloids and derivative.

Principal component analysis (PCA) was performed on six quality control (QC) samples and fourteen experimental samples (seven per group). The QC samples showed tight clustering in both ionization modes (Figure 4A,B), demonstrating a high reproducibility and assay stability. In negative ion mode, the first two principal components (PC1 and PC2) explained 38.5% and 21.4% of the total variance, respectively. In positive ion mode, PC1 and PC2 explained 42.3% and 17.1% of the variance. The PCA score plots revealed a clear separation between the CON and YC groups, with good intra-group sample aggregation, confirming the reliability of the data for subsequent analysis.

To enhance the discrimination between groups and identify metabolites responsible for the observed separation, orthogonal partial least squares discriminant analysis (OPLS-DA) was employed. This method effectively filters out orthogonal signals unrelated to class separation. The OPLS-DA model parameters (Figure 4C) indicated a good explanatory power (R2X, R2Y) and predictive ability (Q2 > 0.6 for all comparisons). A 1000-permutation test was conducted to validate the model and prevent overfitting. The OPLS-DA score plot (Figure 4D) demonstrated a clear separation between the YC and CON groups with tight within-group clustering, confirming the robustness of the model.

Differential metabolites were identified using a combination of variable importance in projection (VIP) values from the OPLS-DA model and statistical significance (*p*-value). Metabolites with VIP > 1 and *p* ≤ 0.05 were considered significantly altered. A volcano plot (Figure 4E) visualized 220 differential metabolites between the YC and CON groups, with 99 upregulated and 121 downregulated in the YC group.

To elucidate the biological implications of these changes, pathway enrichment analysis of the differential metabolites was conducted using KEGG. The results, presented in a bubble diagram (Figure 4F), revealed that the metabolites were significantly enriched in the pyrimidine metabolism pathway (*p* = 0.025).

**Figure 5:**
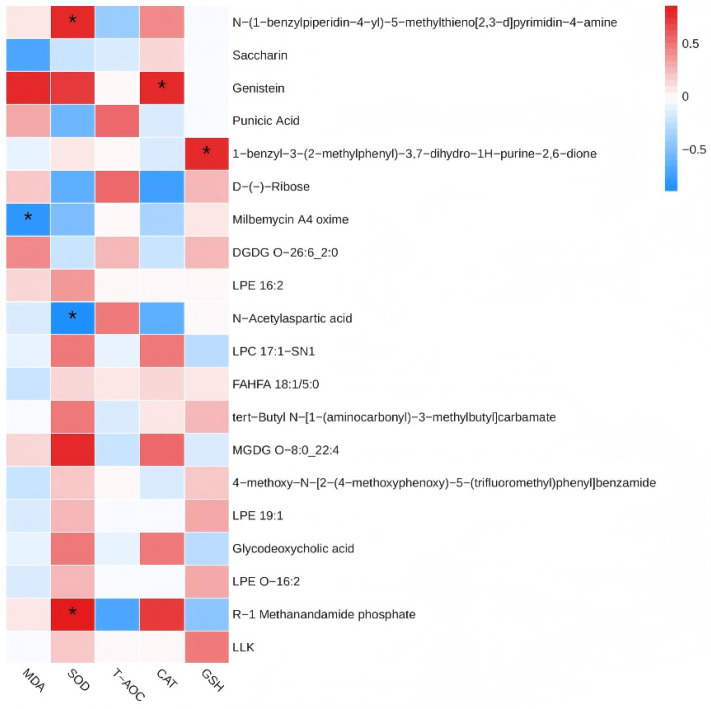
Correlation analysis between differential rumen metabolites and serum antioxidant parameters in dairy cows. Figure 5 shows a heatmap depicting Spearman’s rank correlation coefficients between the top 20 differential metabolites (selected based on variable importance in projection (VIP) scores from the OPLS-DA model) and key serum antioxidant indices: superoxide dismutase (SOD), catalase (CAT), malondialdehyde (MDA), and total antioxidant capacity (T-AOC). Red indicates a positive correlation, and blue indicates a negative correlation. Asterisks denote statistical significance (* *p* < 0.05). Metabolite abbreviations are listed on the left, with antioxidant parameters at the bottom. GEN, genistein

To investigate potential links between ruminal metabolic shifts and the observed systemic antioxidant enhancement, Spearman correlation analysis was conducted between the top 20 VIP-ranked differential metabolites and key serum antioxidant parameters (SOD, CAT, MDA, T-AOC). Several significant correlations were identified (Figure 5). Notably, the ruminal concentration of genistein, a prominent isoflavone, showed a significant positive correlation with serum CAT activity (r = 0.56, *p* < 0.05). This suggests that YC-induced increases in ruminal genistein may potentiate the host’s catalase-mediated antioxidant defense. Furthermore, N-acetylaspartic acid was negatively correlated with MDA levels (r = −0.48, *p* < 0.05), implying a potential role in mitigating lipid peroxidation. Positive and negative correlations were also observed between R-1 Methanandamide phosphate (r = 0.51, *p* < 0.05) and Milbemycin A4 oxime (r = −0.44, *p* < 0.05), respectively, with SOD activity.

These correlation patterns suggest that modulation of the ruminal metabolome, particularly through metabolites like genistein, is a potential mechanism by which YC supplementation enhances the systemic antioxidant status in late-lactation dairy cows.

## 4. Discussion

### 4.1. Effects of YC on Nutrient Digestibility and Lactation Performance

The apparent digestibility of nutrients serves as a critical indicator of an animal’s capacity to digest and absorb dietary components. Our findings demonstrate that dietary supplementation with yeast culture (YC) significantly enhanced the apparent digestibility of dry matter (DM), crude protein (CP), ether extract (EE), neutral detergent fiber (NDF), and acid detergent fiber (ADF). These results align with previous studies reporting improved nutrient digestibility in ruminants supplemented with yeast-based additives, such as active dry yeast or Saccharomyces cerevisiae preparations [17,28]. The enhanced digestibility likely stems from YC’s ability to stimulate ruminal microbial activity, thereby facilitating more efficient fiber breakdown and nutrient release.

Consistent with the improved nutrient utilization, YC supplementation significantly increased average daily milk yield. This finding corroborates numerous reports indicating that yeast additives can enhance milk production in dairy cows, including during the late-lactation period [28,29]. While some studies have noted increases in milk fat and protein yield [30], our experiment specifically observed a significant rise in milk lactose percentage and a reduction in somatic cell count, with no significant changes in milk fat percentage, milk protein percentage, or milk urea nitrogen. This pattern is similar to the results reported by Nocek et al. [30]. The variability in milk composition responses across studies can be attributed to differences in experimental conditions, including feeding systems, animal physiology, yeast dosage, and basal diet composition [17].

The 3.1% increase in DMI (*p* = 0.02) observed with YC supplementation falls within the 2–5% range documented in meta-analyses [28]. This incremental improvement in intake, alongside enhanced digestibility, contributed to the higher milk yield noted in cows receiving YC.

### 4.2. Enhancement of Systemic Antioxidant Status and Metabolic Health by YC

Blood parameters provide a window into systemic metabolic and health status. Oxidative stress, resulting from an imbalance between free radical production and antioxidant defenses, can impair animal health and productivity. In this study, YC supplementation significantly elevated serum levels of superoxide dismutase (SOD) and total antioxidant capacity (T-AOC), indicating a bolstered endogenous antioxidant defense system. This is consistent with reports that yeast-derived components, such as β-glucans, can enhance antioxidant enzyme activities [31,32]. The reduction in the lipid peroxidation marker malondialdehyde (MDA) in other studies [8] further supports the antioxidant potential of YC, although MDA did not change significantly in our trial.

YC also favorably altered key metabolic biomarkers. The significant increase in serum glucose (GLU) concentration suggests enhanced gluconeogenesis or glucose metabolism, a finding previously observed in heat-stressed cows supplemented with active dry yeast [34]. Elevated serum albumin (ALB) and blood urea nitrogen (BUN) levels indicate improved protein absorption and metabolism [35], aligning with the observed increase in CP digestibility. Although alanine aminotransferase (ALT) activity was significantly lower in the YC group (*p* = 0.01), this change (8.5%) was within the normal physiological range for dairy cows (20–40 U/L) and may not have clinical significance. Therefore, we refrain from overinterpreting this finding as evidence of hepatoprotection. Collectively, these hematological improvements correlate with and may underpin the enhanced production performance observed.

### 4.3. Modulation of Rumen Fermentation Characteristics

The rumen is central to ruminant digestion, and its fermentation profile directly impacts host nutrition. YC supplementation did not alter ruminal pH, which remained within the optimal range (5.5–7.5) for microbial proliferation. A stable pH between 6.0 and 6.5 is particularly conducive to microbial growth and volatile fatty acid (VFA) production [36].

A key finding was the significant reduction in ruminal ammonia–nitrogen (NH_3_-N) concentration coupled with a significant increase in microbial crude protein (MCP) content. This suggests that YC enhanced the efficiency of nitrogen capture by rumen microbes, converting more dietary and recycled nitrogen into microbial protein rather than losing it as ammonia. This aligns with the established role of yeast in stimulating microbial growth and improving ammonia utilization [38].

YC significantly increased the concentrations of total VFA and its major components: acetate, propionate, and butyrate. Propionate is a primary gluconeogenic precursor, and its increase likely explains the elevated serum GLU levels. The shift towards propionate production, evidenced by a decreased acetate-to-propionate ratio, indicates a change in the fermentation pattern. This shift may result from YC-induced improvements in fiber digestibility, altering the substrate availability for microbial fermentation, or from changes in rumen epithelial absorption rates. The overall stimulation of VFA production provides more energy to the host, supporting the increased milk yield.

The observed increase in microbial crude protein (MCP) and the concurrent reduction in ruminal NH_3_-N suggest the enhanced efficiency of nitrogen utilization. Although direct measurements of duodenal flow were not performed, these findings imply an increased supply of metabolizable protein (MP) to the host. Furthermore, improved ruminal nitrogen capture holds positive environmental implications, as decreased ammonia concentrations may mitigate nitrogen emissions from dairy operations. Interestingly, the elevated serum urea nitrogen (BUN) levels in the YC group suggest that this captured nitrogen was not merely sequestered, but rather actively metabolized and recycled, likely reflecting an accelerated overall nitrogen turnover. While increased BUN can sometimes indicate excess nitrogen intake, the concurrent improvement in ruminal parameters in our study supports the interpretation of enhanced nitrogen turnover rather than inefficient protein utilization.

### 4.4. Reshaping of the Rumen Microbiota

The rumen microbiome’s composition is pivotal for its function. While α-diversity indices (Shannon, Simpson) showed only a trend toward increase (*p* = 0.06), YC supplementation induced significant taxonomic shifts. At the phylum level, we observed a significant increase in Firmicutes and a decrease in Bacteroidota. As Firmicutes are key fiber degraders and major producers of acetate, their enrichment aligns with the observed increases in fiber digestibility and acetate concentration. Conversely, Bacteroidota, proficient in degrading complex carbohydrates and proteins, decreased in relative abundance. This apparent paradox—reduced Bacteroidota yet improved overall digestibility—may be explained by a more profound intestinal digestion or by functional changes within the microbial community that are not fully captured by taxonomy alone.

Notably, YC increased the relative abundance of archaea (primarily Methanobacteriota) and unclassified viruses. The role of methanogens in maintaining ruminal redox balance is well-known, but the ecological significance of the viral community (virome) in the rumen remains largely unexplored and warrants future investigation.

At the genus level, YC supplementation significantly reduced the abundance of Prevotella and UBA4372 (both within Bacteroidota), consistent with the phylum-level shift. However, Linear Discriminant Analysis Effect Size (LEfSe) identified several bacterial genera enriched in the YC group, including Butyricicoccus (a butyrate producer with anti-inflammatory properties) and Succinivibrio (a cellulolytic bacterium) [38,39]. These specific enrichments may contribute to improved fiber degradation and host health.

### 4.5. Functional Shifts in the Rumen Metagenome and Metabolome

Functional analysis of the rumen metagenome via the CAZy database revealed that YC supplementation significantly reduced the overall abundance of carbohydrate-active enzymes (CAZymes), specifically polysaccharide lyases (PLs) and carbohydrate esterases (CEs). Although this reduction may appear counterintuitive in light of the improved fiber digestibility, it might reflect a shift in the microbial community composition toward species possessing more efficient enzymatic systems rather than a mere decline in absolute enzyme production. Alternatively, these results suggest that fiber degradation may be primarily driven by a subset of highly active microbial populations rather than the collective metabolic output of the entire community. Future investigations employing metatranscriptomic analyses are warranted to elucidate the underlying functional dynamics and clarify the metabolic mechanisms contributing to these observations.

Metabolomic analysis provided a direct readout of ruminal metabolic activity. The most significant finding was the enrichment of differential metabolites in the pyrimidine metabolism pathway. Pyrimidines are essential for nucleic acid synthesis, and their altered metabolism may reflect changes in microbial growth rates and turnover. Among the top upregulated metabolites in the YC group were genistein, a potent isoflavone antioxidant, and punicic acid, a conjugated fatty acid known for its anti-inflammatory properties [39,40,41,42,43,44]. Although genistein is not a direct constituent of the yeast culture, it is presumably derived from dietary soybean meal. The elevated ruminal concentration of genistein in YC-fed cows likely stems from the microbial biotransformation of dietary precursors, potentially facilitated by YC-induced shifts in the ruminal microbial community. Furthermore, this metabolite may contribute to the observed improvements in systemic antioxidant status, as evidenced by its positive correlation with serum catalase (CAT) activity (r = 0.56, *p* < 0.05).

### 4.6. Integrating Microbiome and Metabolome Data: A Proposed Mechanism for Antioxidant Enhancement

The correlation analysis between rumen metabolites and serum antioxidant parameters offers a compelling link between local rumen changes and systemic effects. The strong positive correlation between ruminal genistein and serum catalase (CAT) activity (r = 0.56, *p* < 0.05) is particularly noteworthy. Genistein is a well-documented antioxidant that can upregulate endogenous antioxidant enzymes. Our data suggest that YC supplementation elevates ruminal genistein levels, which may then be absorbed and contribute to the enhanced systemic antioxidant defense observed. This establishes genistein as a potential key mediator linking YC-induced rumen metabolic shifts to improved host antioxidant status.

### 4.7. Synthesis: A Holistic Mechanistic Framework for YC Action

Integrating our multi-omics and physiological data, we propose a coherent mechanistic framework to explain the benefits of YC supplementation in late-lactation dairy cows (Graphical Abstract). The process begins with YC as the input, which directly introduces bioactive components and acts as a prebiotic to modulate the rumen ecosystem.

This input drives a microbial ecological and functional restructuring. Taxonomically, it promotes a shift towards a fibrolytic-oriented community, characterized by an increased Firmicutes/Bacteroidota ratio and the enrichment of specific genera like Succinivibrio. Functionally, this restructured community exhibits altered CAZyme profiles, indicating a potential optimization of carbohydrate degradation strategies. Concurrently, microbial metabolic networks are rewired, as evidenced by the significant enrichment of metabolites in the pyrimidine metabolism pathway, reflecting changes in microbial nucleic acid synthesis and turnover.

The restructured microbiome and its altered metabolism lead to the production of key functional molecules. Most notably, the ruminal concentration of genistein is significantly increased. This bioactive isoflavone, along with other elevated metabolites like punicic acid, represents a critical link between rumen events and host physiology.

Finally, these events culminate in improved host physiological and production outputs. The microbial shift enhances fiber digestion and propionate production, directly boosting energy availability and gluconeogenesis, thereby supporting increased milk yield. Simultaneously, the absorbed ruminal metabolites, particularly genistein, enhance the host’s systemic antioxidant capacity (e.g., increased CAT activity) and improve metabolic health (e.g., liver function). The synergistic effect of improved energy–protein metabolism and enhanced systemic health underpins the overall enhancement in production performance.

In conclusion, YC supplementation exerts its effects not through a single pathway, but through an integrated series of events in the rumen microbiome and metabolome that collectively enhance nutrient utilization and host health. This study highlights genistein as a novel potential mediator in yeast-based feed additives and provides a multi-dimensional perspective for understanding how microbial modulators improve ruminant production.

### 4.8. Limitations and Future Directions

This study acknowledges several limitations that warrant consideration. First, while the sample size of seven animals per group was sufficient to detect significant differences in primary production parameters, it may be underpowered for certain high-variability omics measurements, thereby potentially limiting the generalizability of our findings. Second, although significant correlations were observed between ruminal metabolites and serum antioxidant indices, these associations do not inherently establish causality. Future studies employing larger cohorts and targeted intervention experiments are essential to validate the proposed mechanistic pathways. Finally, the absence of detailed compositional data for the yeast culture product—due to commercial confidentiality—precludes direct comparisons with other formulations and, to some extent, constrains a comprehensive mechanistic interpretation of its specific effects.

## 5. Conclusions

This study demonstrates that dietary supplementation with yeast culture (YC) during the late-lactation period enhances production performance in Holstein cows through a multi-faceted mechanism involving improved nutrient digestibility, optimized rumen fermentation, and a comprehensive modulation of the ruminal microbiota and metabolome. YC supplementation significantly increased dry matter intake (+3.1%), nutrient digestibility, and milk yield (+5.75 kg/d), while concurrently reducing the somatic cell count. These phenotypic improvements were underpinned by enhanced ruminal function, characterized by elevated volatile fatty acid production, greater microbial protein synthesis, and augmented systemic antioxidant capacity. Furthermore, integrated multi-omics analysis revealed that YC orchestrates a shift in the ruminal microbial community toward a more fibrolytic profile and enriches bioactive metabolites, such as genistein, which exhibited a positive correlation with improved antioxidant status. Collectively, these findings provide a robust mechanistic framework for utilizing yeast culture as a nutritional strategy to sustain performance in late-lactation dairy cows, while underscoring the potential of rumen-derived metabolites as predictive biomarkers for evaluating the efficacy of dietary feed additives.

## Figures and Tables

**Table 1:** Ingredients and chemical composition of the basal diet (% of dry matter).

Item	Diet
Diet composition	
Corn silage	32.09
Oat hay	2.08
Cottonseed	4.25
Beet pulp	4.96
Flaked corn	15.55
High-moisture corn	7.08
Corn flour	9.70
Bean pulp	10.63
Extruded soybean	4.60
Rapeseed dregs	4.96
Premix ^1^	2.53
H_5_KMgO_4_S	0.19
C16 fat	1.36
Flavoring agent	0.01
Nutrient level	
Crude Prot (%)	16.82
Ether extract (%)	5.89
Crude fiber (%)	52.4
Net energy of lactation (Mcal/kg)	6.92
Phosphorus (%)	0.64
Calcium (%)	1.3
Concentrate:forage ratio	1.65:1

^1^ Premix: VA, 22.5 KIU/kg; VD3, 5.0 KIU/kg; VE, 37.5 IU/kg; VK3, 5.0 mg/kg; Mn, 63.5 mg/kg; Zn, 111.9 mg/kg; Cu, 25.6 mg/kg; Fe, 159.3 mg/kg.

**Table 2:** Effects of dietary yeast culture supplementation on dry matter intake and total-tract apparent digestibility of nutrients in late-lactation dairy cows.

Items	CON ^1^	YC ^1^	SEM ^2^	*p*-Value
Feed intake, kg/d	23.51	24.23 *	0.53	0.02
Digestibility, %				
DM	0.86	0.92 **	0.01	<0.01
CP	0.60	0.80 **	0.02	<0.01
EE	0.58	0.89 **	0.04	<0.01
NDF	0.52	0.72 **	0.03	<0.01
ADF	0.19	0.45 **	0.07	0.01

^1^ Treatments included a basal control diet (CON) and a basal diet with supplemental 50 g/d yeast culture (YC). ^2^ Largest SEM. DM: Dry material. CP: Crude protein. EE: Ether extract. NDF: Neutral detergent fiber. ADF: Acid detergent fiber. * represent significant differences (*p* < 0.05); ** represent highly significant differences (*p* < 0.01).

**Table 3:** Lactation performance of late-lactation dairy cows supplemented with or without yeast culture.

Items	CON ^1^	YC ^1^	SEM ^2^	*p*-Value
Milk yield, kg/d	27.04	32.79 **	5.38	<0.01
Milk composition, %				
Lactose	4.82	5.04 *	0.12	0.05
Fat	3.62	3.99	0.51	0.35
Protein	3.45	3.46	0.13	0.70
SCC ^3^, 10^4^/mL	25.39	24.81	3.14	0.61
Urea nitrogen, mg/dL	12.23	11.49	2.44	0.79

^1^ Treatments included a basal control diet (CON) and a basal diet with supplemental 50 g/d yeast culture (YC). ^2^ Largest SEM. ^3^ SCC: Somatic cell count. * represent significant differences (*p* < 0.05); ** represent highly significant differences (*p* < 0.01).

**Table 4:** Serum antioxidant indices and biochemical parameters in late-lactation dairy cows supplemented with or without yeast culture.

Items	CON ^1^	YC ^1^	SEM ^2^	*p*-Value
Antioxidant capacity				
MDA, μM	2.37	2.80	0.78	0.30
SOD, U/mL	11.32	14.34 **	1.41	<0.01
CAT, U/mL	2.40	3.30 *	0.63	0.02
T-AOC, mM	0.26	0.30 *	0.04	0.05
GSH, μM	9.07	10.34 **	1.98	0.01
Biochemistry				
GLU, mM	3.47	4.32 **	0.22	<0.01
ALB, g/L	34.56	38.56 **	2.71	<0.01
ALT, U/L	35.12	32.12 **	3.85	0.01
AST, U/L	174.69	226.23	65.06	0.57
BUN, mM	4.12	5.68 **	0.42	<0.01

^1^ Treatments included a basal control diet (CON) and a basal diet with supplemental 50 g/d yeast culture (YC). ^2^ Largest SEM. MDA: Malondialdehyde. SOD: Superoxide dismutase. CAT: Catalase. T-AOC: Total antioxidant capacity. GLU: Glucose. ALB: Albumin. ALT: Alanine aminotransferase. AST: Aspartate transaminase. BUN: Blood urea nitrogen. * represent significant differences (*p* < 0.05); ** represent highly significant differences (*p* < 0.01).

**Table 5:** Rumen fermentation parameters of late-lactation dairy cows supplemented with or without yeast culture.

Items	CON ^1^	YC ^1^	SEM ^2^	*p*-Value
Ruminal pH	6.21	5.79	0.30	0.10
NH_3_-N, mg/dL	20.11	15.94 **	2.02	<0.01
Microbial protein, mg/mL	0.94	1.19 **	0.12	<0.01
Lactic acid, U/L	0.94	0.87	0.64	0.17
VFA, mM				
Acetate, mM	63.55	70.78 *	3.15	0.02
Propionate, mM	24.22	35.77 *	6.02	0.02
Isobutyrate, mM	1.02	0.68	0.25	0.06
Butyrate, mM	14.28	17.94 **	1.30	<0.01
Isovalerate, mM	1.49	1.39	0.27	0.55
Valerate, mM	1.68	2.03	0.48	0.23
Acetate: Propionate	2.63	2.04 *	0.35	0.02
Total VFA, mM	106.24	128.58 **	5.66	0.01

^1^ Treatments included a basal control diet (CON) and a basal diet with supplemental 50 g/d yeast culture (YC). ^2^ Largest SEM. * represent significant differences (*p* < 0.05); ** represent highly significant differences (*p* < 0.01).

**Table 6:** Relative abundance of rumen bacteria at the phylum and genus levels in late-lactation dairy cows supplemented with or without yeast culture.

Items	CON ^1^	YC ^1^	SEM ^2^	*p*-Value
Phylum				
Bacteroidota	63.74	51.94 **	6.98	0.01
Firmicutes	21.13	30.03 **	5.83	0.01
Proteobacteria	1.56	2.11	0.55	0.08
Methanobacteriota	0.56	0.81 *	0.22	0.03
Patescibacteria	0.58	0.42 **	0.10	0.01
Spirochaetota	0.74	0.55 **	0.09	0.01
Verrucomicrobiota	0.67	0.40 *	0.23	0.05
Viruses_norank	0.68	1.18 *	0.47	0.03
Genes				
Prevotella	53.63	39.49 **	4.49	<0.01
Cryptobacteroides	3.37	2.63	0.28	0.09
Limimorpha	1.65	0.76 **	0.27	0.01
UBA4372	2.21	1.44 **	0.33	<0.01
UBA1711	2.18	1.67	0.49	0.07
RF16	1.26	0.41 **	0.23	0.00
UBA4334	1.11	0.63 **	0.13	<0.01

^1^ Treatments included a basal control diet (CON) and a basal diet with supplemental 50 g/d yeast culture (YC). ^2^ Largest SEM. * represent significant differences (*p* < 0.05); ** represent highly significant differences (*p* < 0.01).

## Data Availability

The original contributions presented in this study are included in the article. Further inquiries can be directed to the corresponding author.

## Abbreviations

YC	Yeast culture
KEGG	Kyoto Encyclopedia of Genes and Genomes
GHs	Glycoside hydrolases
CAZys	Carbohydrate-active enzymes
DM	Dry matter
CP	Crude protein
EE	Ether extract
NDF	Neutral detergent fiber
ADF	Acid detergent fiber
AIA	Acid insoluble ash
MDA	Malondialdehyde
SOD	Superoxide dismutase
CAT	Catalase
T-AOC	Total antioxidant capacity
GSH	Reduced glutathione
GLU	Glucose
ALB	Albumin
ALT	Alanine aminotransferase
BUN	Blood urea nitrogen
AST	Aspartate transaminase
VFA	Volatile fatty acids
NH_3_-N	Ammonia nitrogen
MCP	Microbial crude protein
